# The data of establishing a three-dimensional culture system for *in vitro* recapitulation and mechanism exploration of tumor satellite formation during cancer cell transition

**DOI:** 10.1016/j.dib.2017.09.053

**Published:** 2017-09-28

**Authors:** Chun-Nan Chen, You-Tzung Chen, Tsung-Lin Yang

**Affiliations:** a Graduate Institute of Clinical Medicine, National Taiwan University College of Medicine, Taipei, Taiwan; b Department of Otolaryngology, National Taiwan University Hospital and National Taiwan University College of Medicine, Taipei, Taiwan; c Graduate Institute of Medical Genomics and Proteomics, National Taiwan University College of Medicine, Taipei, Taiwan; d Research Center for Developmental Biology and Regenerative Medicine, National Taiwan University, Taipei, Taiwan

**Keywords:** Three-dimensional culture system, *in vitro* recapitulation, Tumor satellites, Cancer, Transition

## Abstract

Tumor satellite formation is an indicator of cancer invasiveness and correlates with recurrence, metastasis, and poorer prognosis. By analyzing pathological specimens, tumor satellites formed at the tumor-host interface reflect the phenomena of epithelial-mesenchymal transition. It is impossible to reveal the dynamic processes and the decisive factors of tumor satellite formation using clinicopathological approaches alone. Therefore, establishment of an *in vitro* system to monitor the phenomena is important to explicitly elucidate underlying mechanisms. In this study, we explored the feasibility of creating an *in vitro* three-dimensional collagen culture system to recapitulate the process of tumor satellite formation. This data presented here are referred to the research article (Chen et al., 2017) [1]. Using this model, the dynamic process of tumor satellite formation could be recapitulated in different types of human cancer cells. Induced by calcium deprivation, the treated cells increased the incidence and migratory distance of tumor satellites. E-cadherin internalization and invadopodia formation were enhanced by calcium deprivation and were associated with cellular dynamic change during tumor satellite formation. The data confirmed the utility of this culture system to recapitulate dynamic cellular alteration and to explore the potential mechanisms of tumor satellite formation.

**Specifications Table**

**Table:** 

Subject area	*Biology; Biomaterials*
More specific subject area	*An inducible three-dimensional in vitro culture system for recapitulating tumor satellite formation of cancer*
Type of data	*Figures and Charts*
How data was acquired	*An in vitro three-dimensional collagen culture system was established for culturing cancer cells. Tumor satellite formation was induced by calcium deprivation. The morphology, cellular features, biological behaviors, and expression of specific markers of tumor satellites were recorded for comparison. The phenotypes and characteristics of tumor cells were analyzed.*
Data format	*Raw and analyzed data*
Experimental factors	*The three-dimensional collagen scaffold and low extracellular calcium concentration were used to induce tumor satellite formation. The results of different types of cancer cells were tested and compared.*
Experimental features	*Induction of tumor satellite formation in the in vitro culture system was determined by image recording and quantitative analyses of cellular features and behaviors.*
Data source location	*The National Taiwan University, Taipei, Taiwan*
Data accessibility	*Data is available within this article*

**Value of the data**

1. Establishment of a three-dimensional culture system serves as the standard experimental platform for efficient induction of tumor satellite formation of cancer.
2. The data allow other researchers to investigate tumor cell behaviors in the biomimetic 3D collagen system, and explore the underlying mechanism accounting for cancer cell transition.
3. The data show the feasible way to monitor dynamic process of epithelial-mesenchymal transition during tumor satellite formation.
4. The data demonstrate the methodology of changing cancer cells by regulating extracellular calcium

## 1 Data

### 1.1 Data

The dataset of this paper provides information related to the article “Application of three-dimensional collagen scaffolds to recapitulate and monitor the dynamics of epithelial mesenchymal transition during tumor satellite formation of head and neck cancer” [1].

### 1.2 Comparison of the parameters of tumor satellites in different HNSCC cell lines in the 3D collagen scaffolds without calcium deprivation

**Fig. 1:**
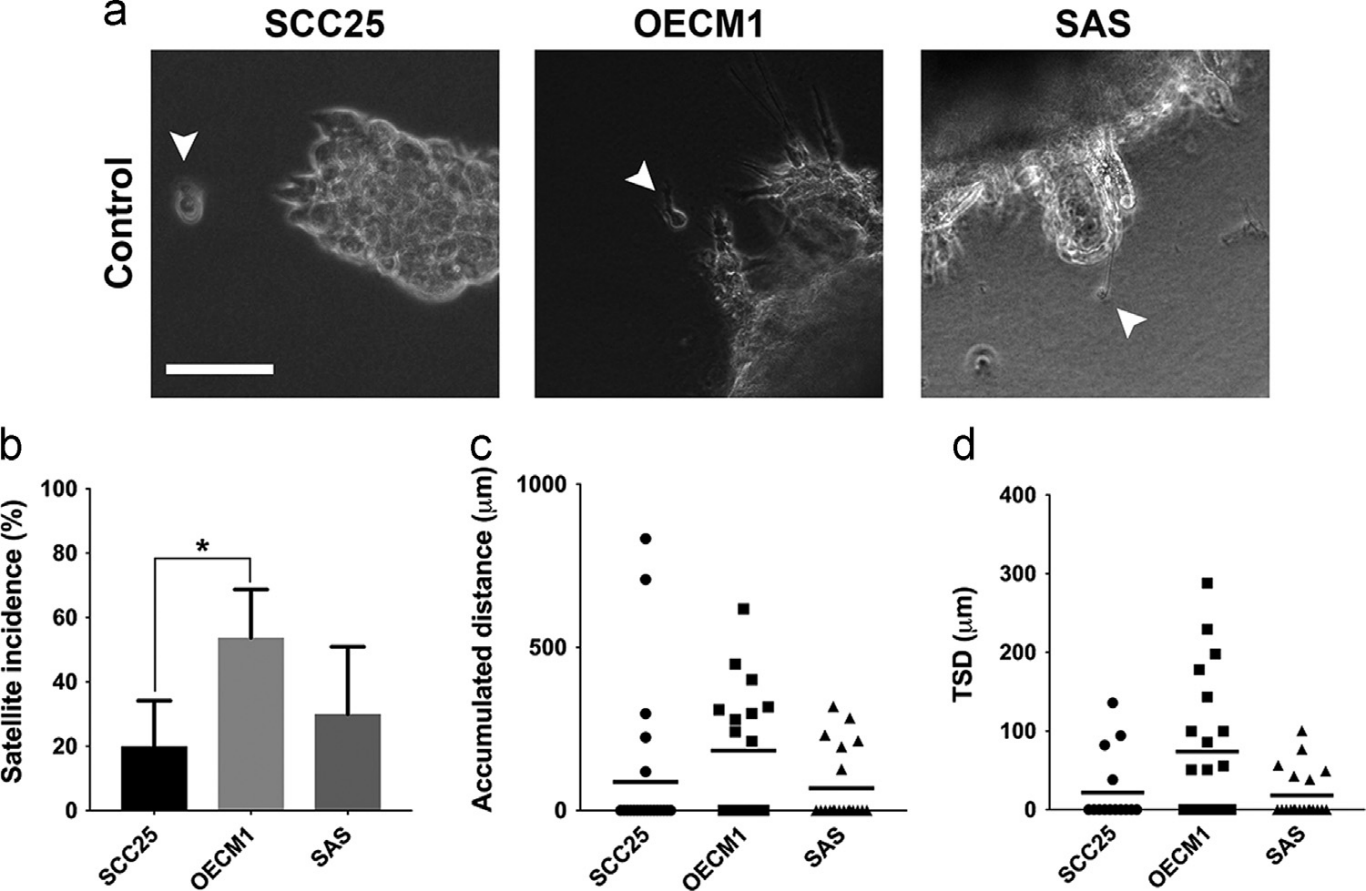
Comparison of the parameters of tumor satellites in distinct HNSCC cell lines in the 3D collagen culture system without calcium deprivation. (a) Tumor satellite formation in SCC25, OECM1 and SAS cells in the 3D collagen culture system without calcium deprivation. (scale bar: 100 μm). (b) The incidences of tumor satellite formation in all HNSCC cell lines (*p < 0.05). (c) The accumulated distances of satellite cells in all HNSCC cell lines. (d) Tumor satellite distance (TSD) in all HNSCC cell lines.

The background and characteristics of the cell lines of head and neck squamous cell carcinoma (HNSCC) were summarized (Table 1). When they were cultured in the 3D system with normal calcium concentrations, the incidence of tumor satellite formation was the highest in OECM1 cells, followed by SAS cells and SCC25 cells (Fig. 1a, b). Though there were no significant differences among the three cell lines regarding the accumulated distance and TSD, the OECM1 cells still demonstrated longer accumulated distances and TSD than others (Fig. 1c, d). The tumor satellite incidence of SCC25 cells was significantly less than OECM1 cells (Fig. 1b). Compatible with the intrinsic biological characteristics, SCC25 cells cultured in the 3D collagen scaffold were less invasive than others.

**Table 1:** The biological background of SCC25, OECM1, and SAS cell lines.

		**SCC25**	**OECM1**	**SAS**	**Refs.**
**Origin**		Tongue	Gingiva	Tongue	[8], [9], [10], [26]
**HPV status**					
HPV-16		−	−	−	[27]
HPV-18		−	−	+	[27]
**Basal invasive capacity**		+	++	+++	[9], [28], [29]
				
**Genetic background**					
	*TP53*	+	+	WT	[30], [31]
	*CDKN2A* (p16)	+	+	+	[30], [32], [33]
				
**Critical proteins**					
LOX		++	++	+	[9]
Tid1		+	+	−	[10]
CK1ε		++	+	N/A	[29]
SIRT1		+	+	N/A	[26]
				
**Tumor suppressor miRNAs**					
	miR-10b	+++	+	+	[27]
	miR-196a	+++	+	+	[27]
	miR-491-5p	+	++	++	[34]
	miR-410	+	+	N/A	[35]
	miR-99a	+	+	N/A	[35]
	miR-21	+	N/A	N/A	[35]
				
**Oncogenic miRNA**					
	miR-31	N/A	+	+	[35]
	miR-146a	N/A	+	+	[36]
	miR-187	N/A	+	+	[37]

WT: wild type; N/A: not available.

### 1.3 Induction of tumor satellite formation in the HNSCC cell lines with low extracellular calcium concentrations

**Fig. 2:**
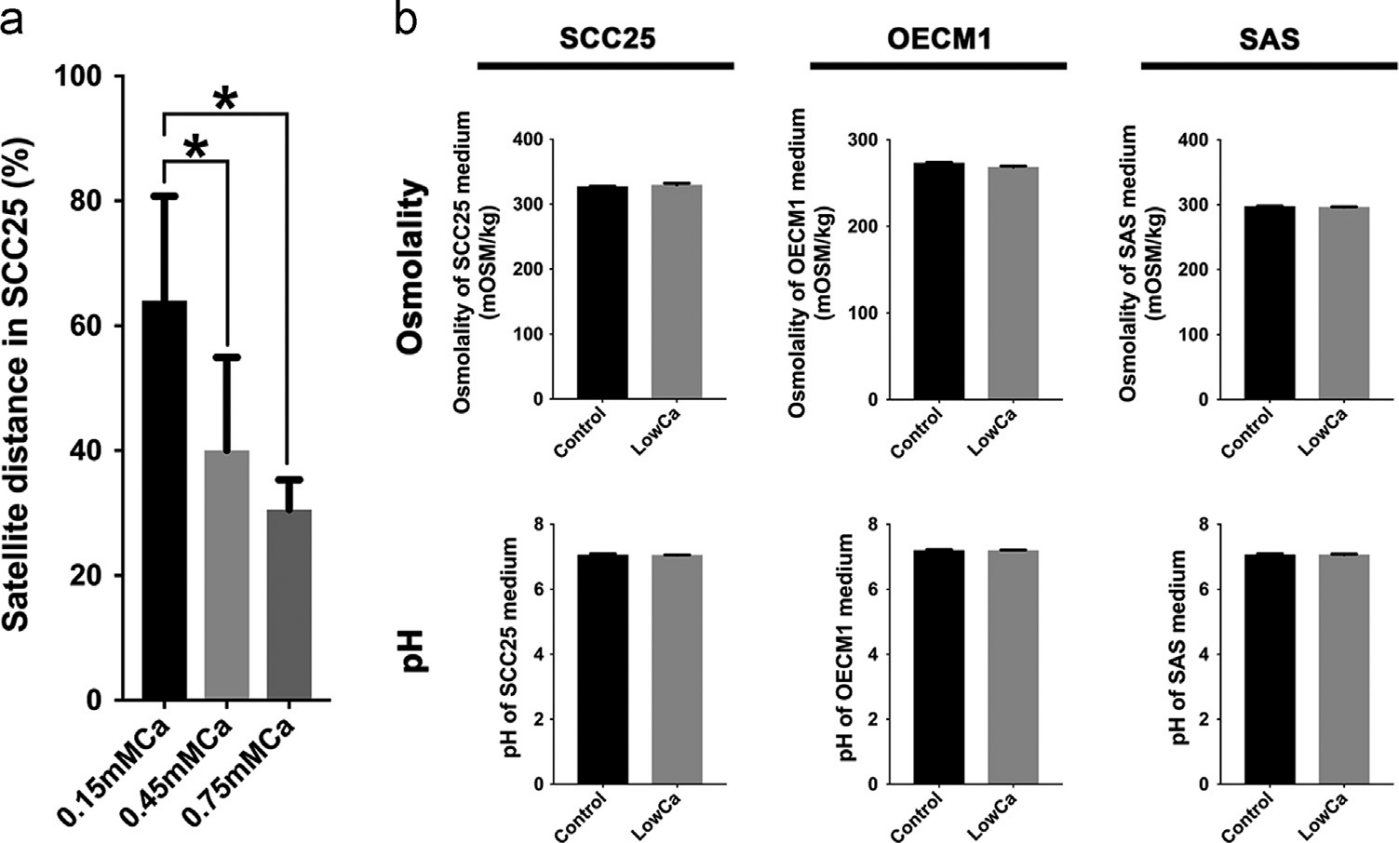
Characteristics of low calcium media used for the tumor satellite experiments of HNSCC cells. (a) SCC25 cells showed a higher incidence of tumor satellites in the medium with lower calcium concentrations. (b) No significant change of osmolality and pH value of the media in all 3 cell lines of the control and the low calcium group (LowCa, 0.15 mM [Ca^2+^]).

**Fig. 3:**
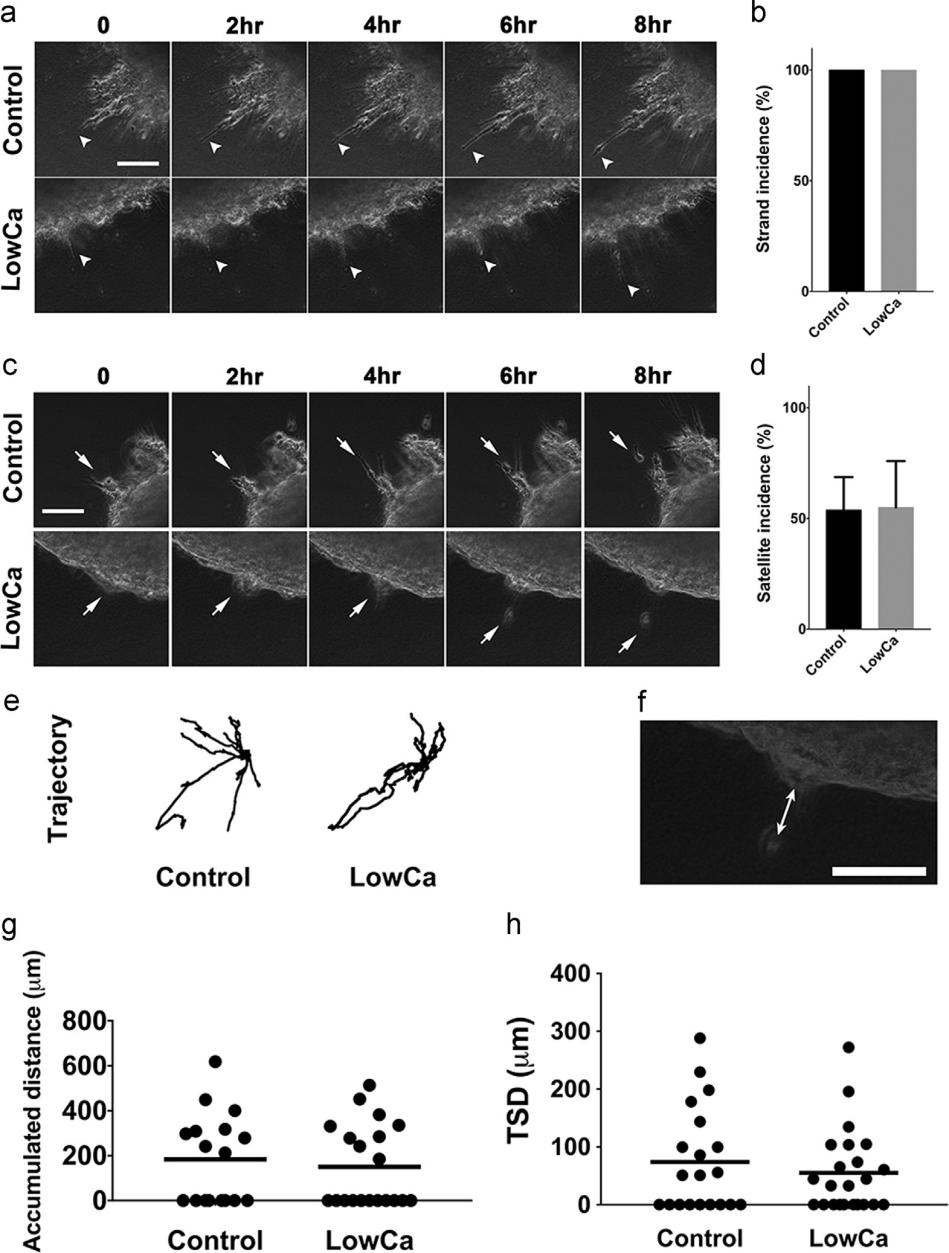
Tumor satellite formation in a three-dimensional collagen culture system with or without reduced extracellular calcium concentrations. (a) Cancer cells (OECM1) grew with the strand pattern (arrowheads) were found in both the control and LowCa environments. (Interval of images: 2 h, scale bar: 100 μm). (b) The incidence of cancer cell growth with the strand pattern was 100% in both groups. (c) Formation of tumor satellites (arrows) in both control and experimental groups. (Interval of images: 2 h, scale bar: 100 μm). (d) The quantitative results of the incidence of tumor satellite formation in both groups. (e) Analyses of trajectories of the satellite cells in both groups. (f) Tumor satellite distance (TSD) is defined as the distance between the parental tumor and the satellite cells. (the arrow with double heads, scale bar: 100 μm). (g) Comparison of accumulated distances of the satellite cells in both groups. (h) Comparison of TSD in both groups. (LowCa: low calcium concentration).

**Fig. 4:**
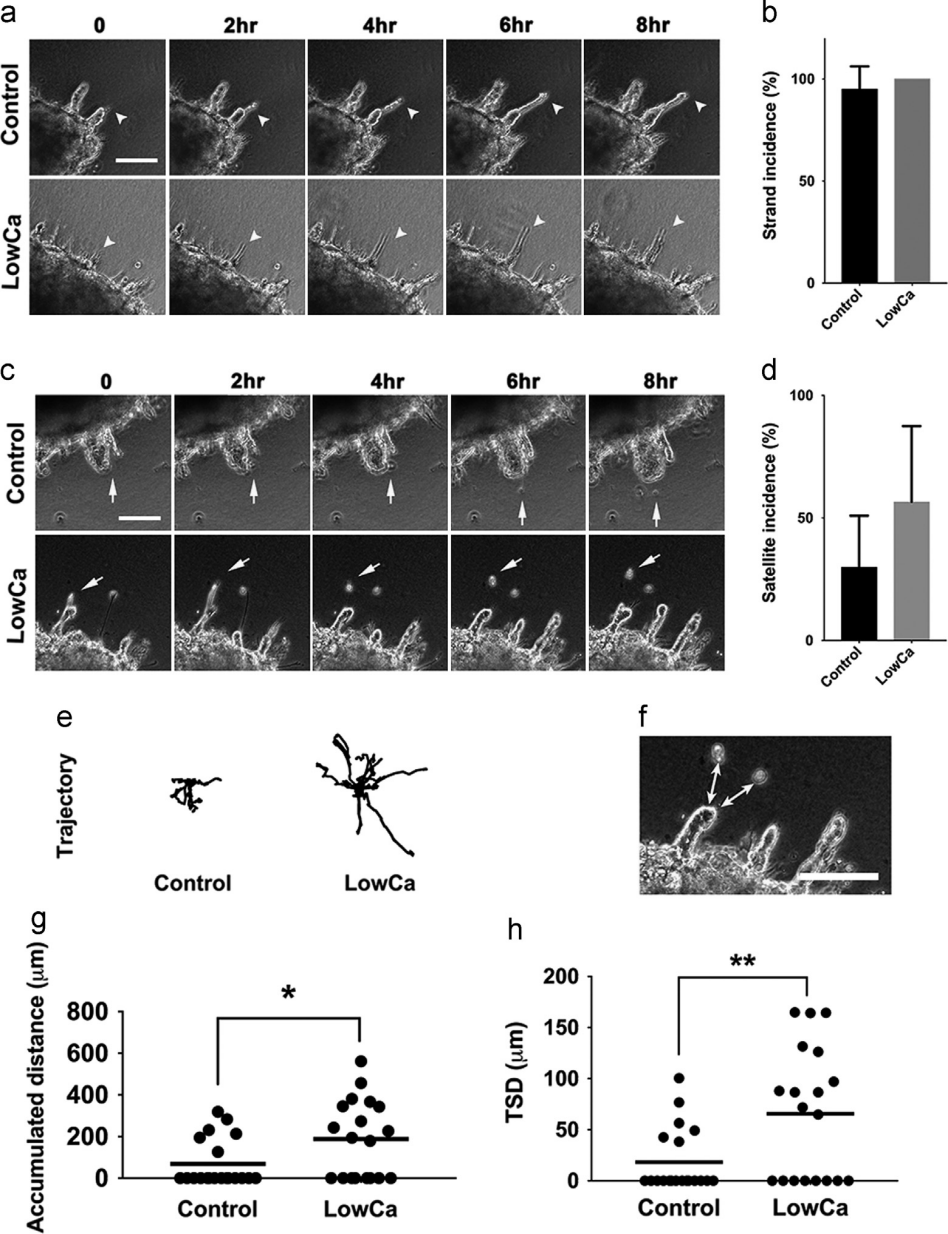
Tumor satellite formation in a three-dimensional collagen culture system with or without reduced extracellular calcium concentrations. (a) Cancer cells (SAS) grew with the strand pattern (arrowhead) were found in both the control and LowCa environments. (Interval of images: 2 h, scale bar: 100 μm). (b) The incidence of cancer cell growth with the strand pattern in both groups. (c) Formation of tumor satellites (arrow) in both control and experimental groups. (Interval of images: 2 h, scale bar: 100 μm). (d) The quantitative results of the incidence of tumor satellite formation in both groups. (e) The analyses of trajectories of the satellite cells in both groups. (f) Tumor satellite distance (TSD) is defined as the distance between the parental tumor and the satellite cells. (the arrow with double heads, scale bar: 100 μm) (g) Comparison of accumulated distances of satellite cells in both groups. (h) Comparison of TSD in both groups. (LowCa: low calcium concentration; *p<0.05; **p<0.01).

Low extracellular calcium concentrations had been reported to range from 0.09 to 0.5 mM in relative to 1.05 mM in normal media [2], [3]. Different concentrations of LowCa media were prepared by serial titration within the ranges. It was found that the experiments with 0.15 mM Ca had the best results of tumor satellite formation (Fig. 2a). 0.15 mM Ca was therefore designed as the low calcium concentration in the following experiments. No alteration of osmolality and pH was found when calcium concentrations were adjusted in the indicated culture medium for each cell line (Fig. 2b). Our data demonstrated the incidence of tumor satellite formation was enhanced in SCC25 cells by LowCa [1]. For OECM1 and SAS cells, no significant enhancement of tumor satellite formation was induced by LowCa (Fig. 3, Fig. 4). When OECM1 cells were treated by LowCa, no significant changes of satellite incidence, accumulated distance, or TSD were found (Fig. 3d, f, h). On the contrary, LowCa increased accumulated distances and TSD of SAS cells in the 3D culture system (Fig. 4, f, h).

### 1.4 Induction of tumor satellite formation in different types of cancer cell lines in the three-dimensional culture system

**Fig. 5:**
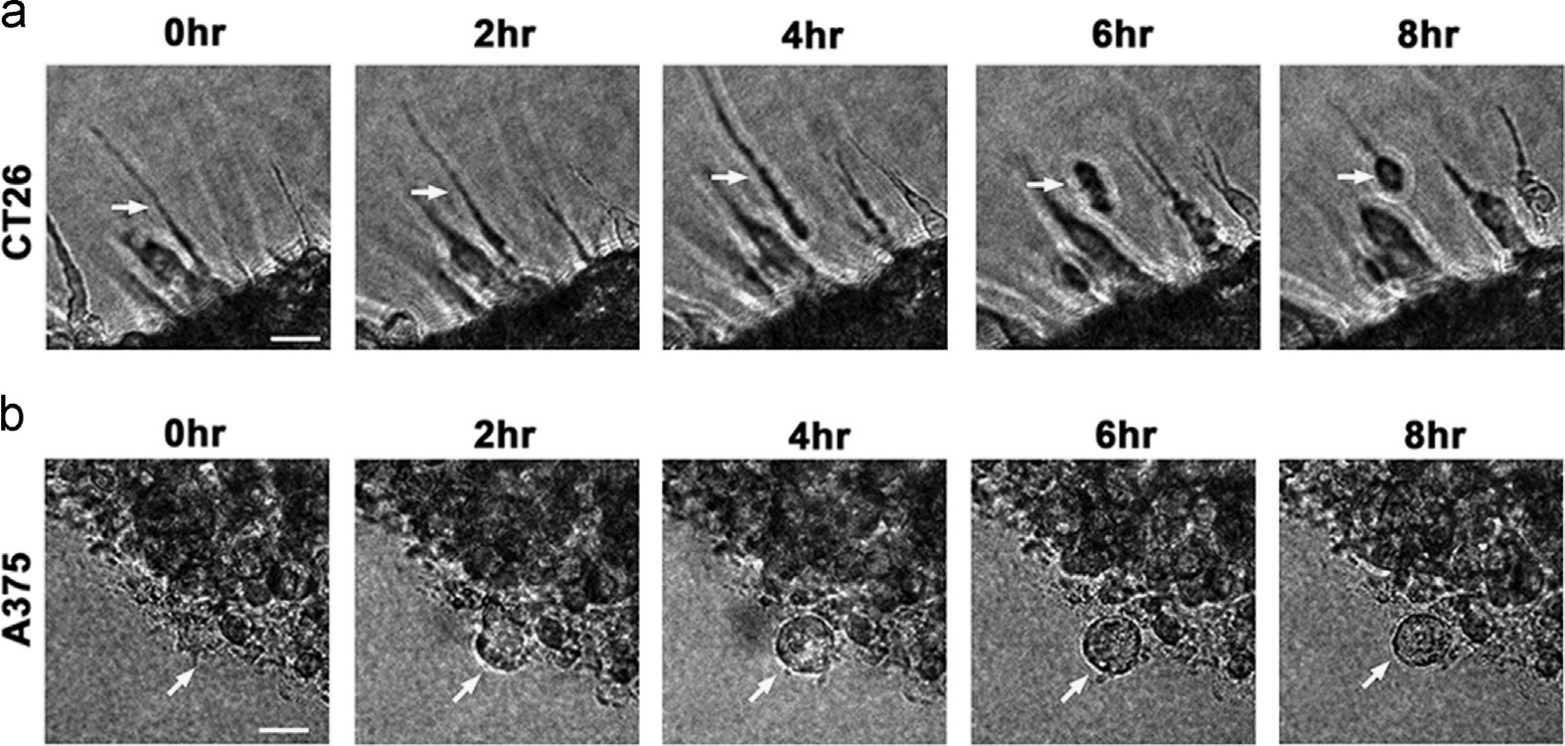
Induction of tumor satellite formation in different types of cancer cell lines in the *in vitro* three-dimensional culture system. Formation of tumor satellites (arrows) could be induced in the *in vitro* three-dimensional culture system for (a) the colorectal cancer cell line (CT26), and (b) the melanoma cell line (A375). (Interval of images: 2 h, scale bar: 50 μm).

In addition to HNSCC, different types of cancer cells were tested to verify the universal utility of inducing tumor satellite formation in this *in vitro* 3D culture system. The colorectal cancer cells (CT26) and the melanoma cells (A375) were selected for culture. Using time-lapse microscopic recording, tumor satellite formation was detected in either CT26 cells (Fig. 5a) or A375 cells (Fig. 5b).

### 1.5 Cell migration, viability, and proliferation of HNSCC cells in 2D culture with calcium deprivation

**Fig. 6:**
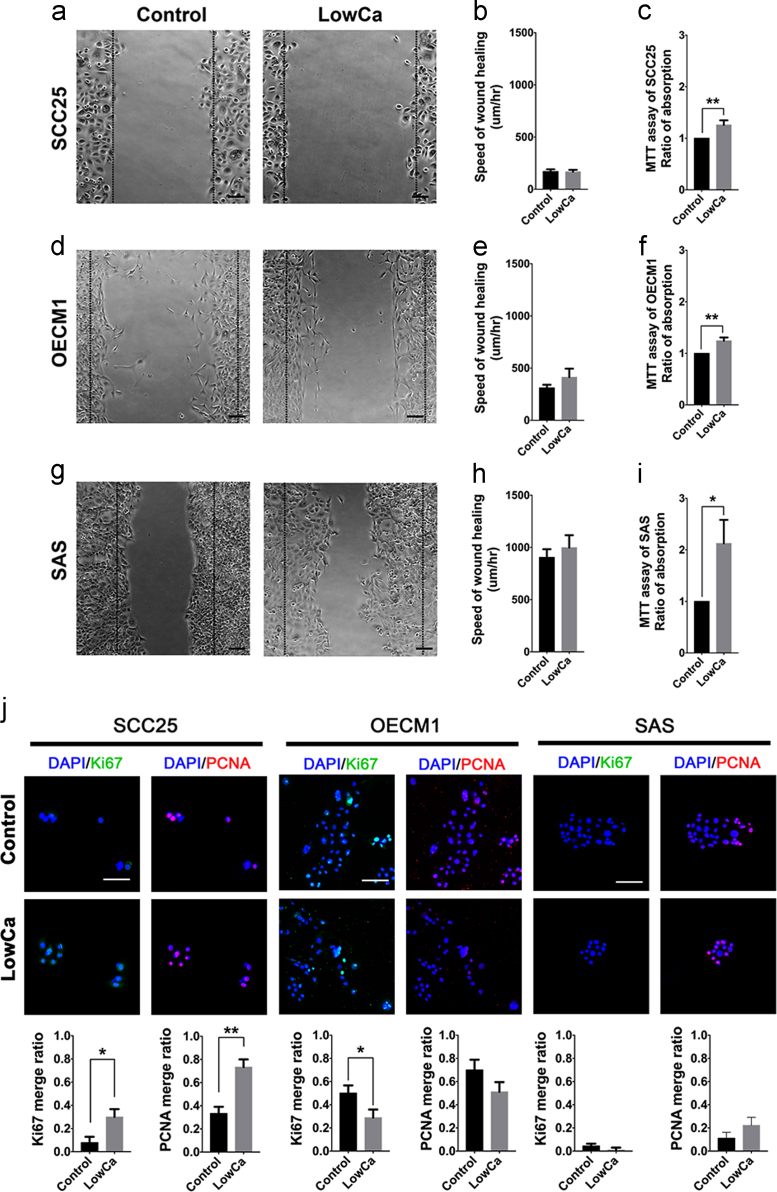
Cell migration, viability, and proliferation of HNSCC cells with calcium deprivation. The wound healing assay of (a) SCC25, (d) OECM1, and (g) SAS cells after cultivated for 20 h with or without low calcium concentrations. The 2 vertical lines indicated the start points of the wound healing assay (Scale bar: 100 μm). Quantitative analyses were demonstrated in (b) SCC25, (e) OECM1, and (h) SAS cells, respectively. The results of MTT assays were shown in (c) SCC25, (f) OECM1, and (i) SAS cells. (j) Proliferative indexes including Ki-67 and PCNA were analyzed in SCC25, OECM1, and SAS cells with or without LowCa concentrations. (LowCa: low calcium concentration; *p<0.05; **p<0.01).

The assays of cell migration, viability and proliferation were applied to evaluate the cells cultivated in the 2D culture system without collagen scaffolds. No change of cellular migration (Fig. 6a, b, d, e, g, h) was found. However, LowCa induced significantly higher metabolic activities in all cell lines than controls (Fig. 6c, f, i). Its impact on proliferation was diverse, showing an increase in the SCC25 cells, a decrease in the OECM1 cells, and no change in the SAS cells (Fig. 6j). It showed that the intrinsic abilities were not completely altered by LowCa independently.

### 1.6 Altered expression of HIF1α and PHD2 in HNSCC cells with calcium deprivation

**Fig. 7:**
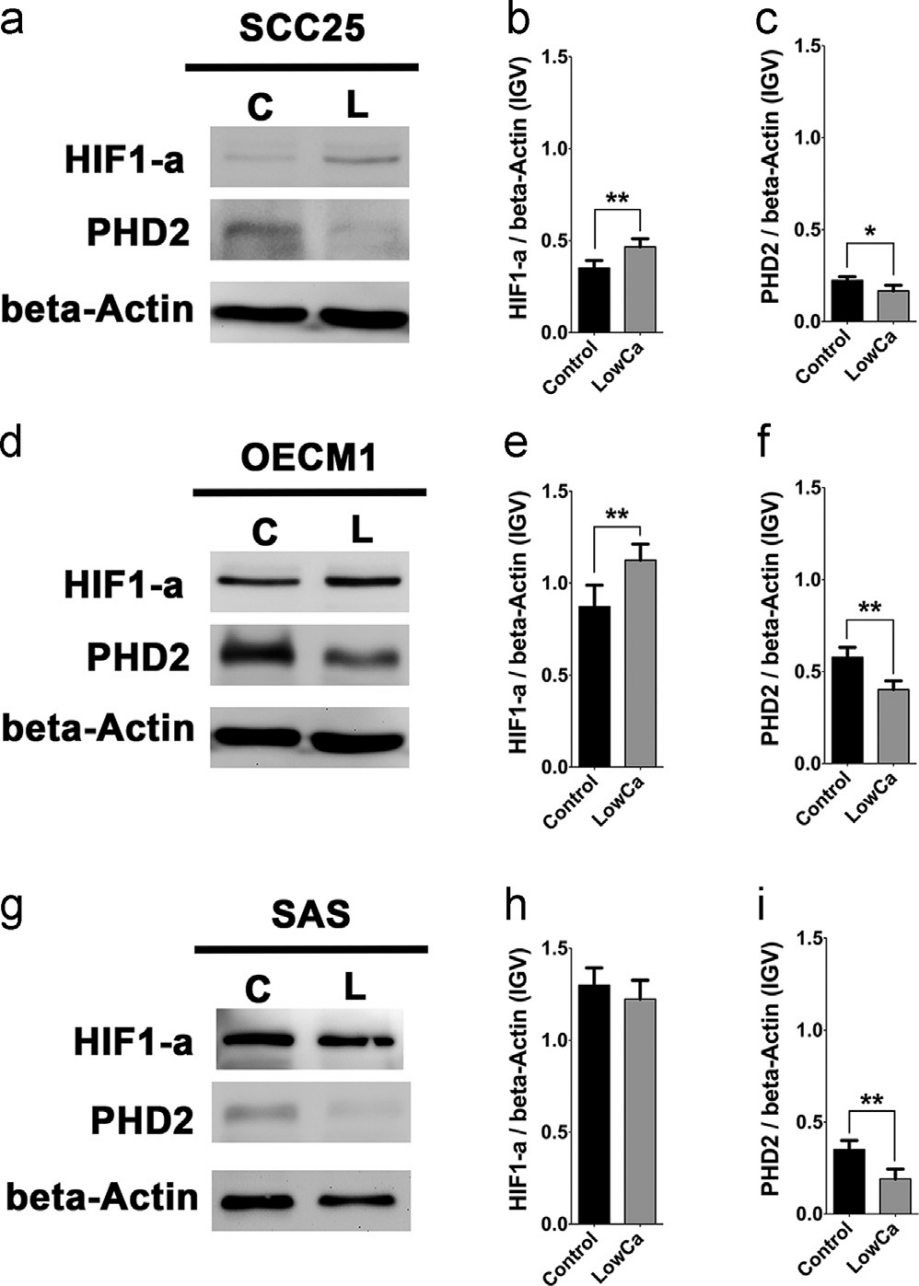
The change of HIF1α and PHD2 in HNSCC cells with calcium deprivation. Western blotting of HIF1α and PHD2 in HNSCC cells in both LowCa (L) and control (C) groups of (a) SCC25, (d) OECM1, and (g) SAS cells. Quantitative changes of HIF1α (b, e, h) and PHD2 (c, f, i) expression in SCC25, OECM1, and SAS cells, respectively. (*p<0.05; **p<0.01).

The western blot was used to confirm the change of HIF1α and PHD2 expression. HIF1α and PHD2 could be detected in all HNSCC cell lines. The expression levels of HIF1α in tested cell lines complied with their oncological aggressiveness listed in Table 1. The SAS and OECM1 cells had higher HIF1α levels than SCC25 cells in the controls (Fig. 7a, d, g)**.** After LowCa treatment, increased HIF1α was found in SCC25 and OECM1 cells, but not in SAS cells (Fig. 7b, e, h). When HIF1α degrading enzyme, prolyl hydroxylase 2 (PHD2), was analyzed, (Fig. 7a, d, g), PHD2 decreased in all cell lines after LowCa treatments (Fig. 7c, f, i). The data demonstrated that HIF1α and PHD2, the decisive factors in epithelial-mesenchymal transition (EMT) of cancer cells, changed their expression levels by calcium deprivation.

### 1.7 Mesenchymal cellular phenotypes were induced by LowCa in HNSCC cells

**Fig. 8:**
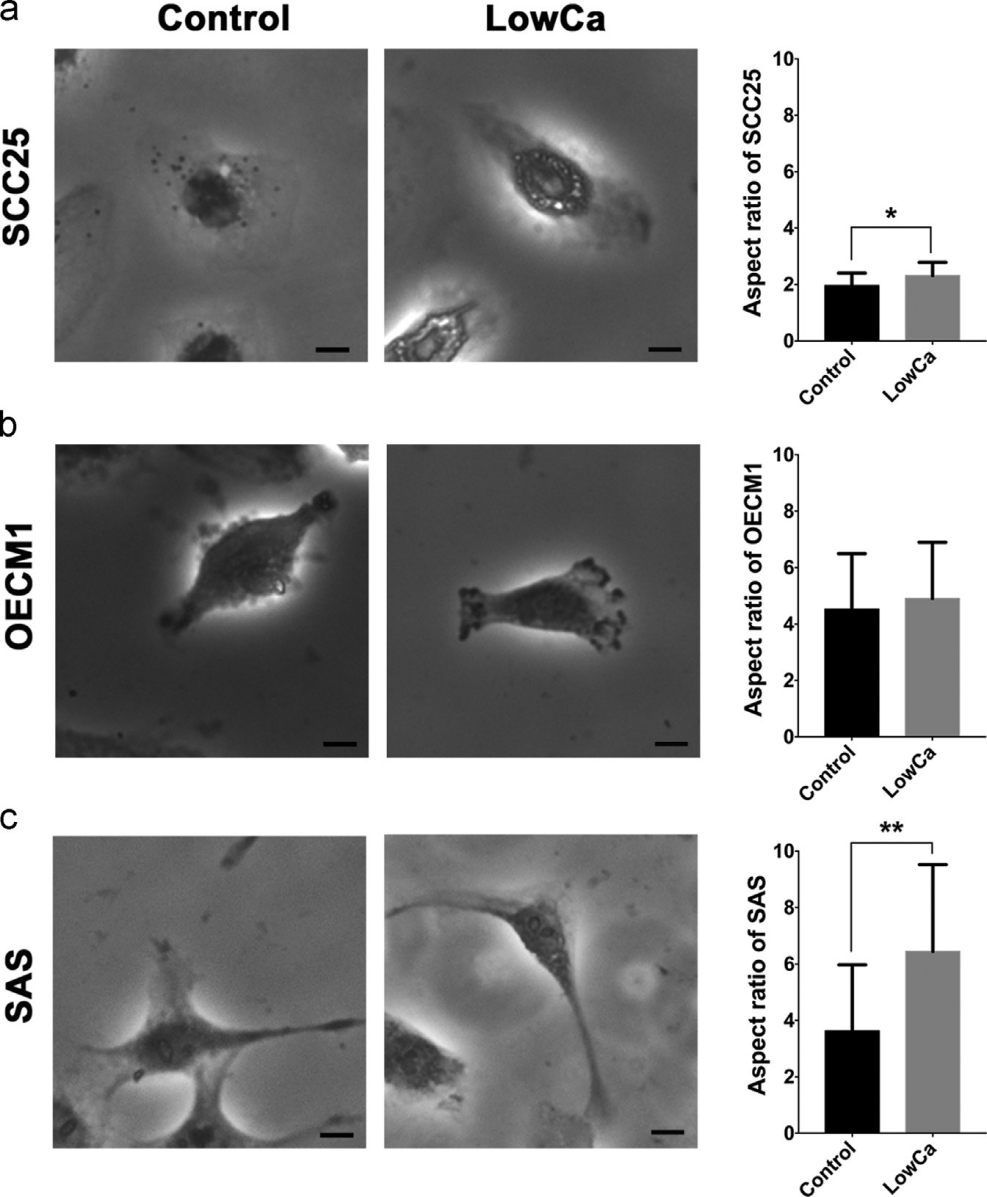
Aspect ratios (AR) of HNSCC cells with calcium deprivation. The change of aspect ratios in the control and the LowCa groups of (a) SCC25, (b) OECM1, and (c) SAS cells. (LowCa: low calcium concentration; *p<0.05; **p<0.01).

To verify the effects of LowCa on cellular phenotypes, the cultured cells of each cell line were recorded for the aspect ratio based on the measurement of the longest side to the shortest side [1]. The mesenchymal phenotypes, which were characterized by high aspect ratios, were observed in all tested cells after calcium deprivation. For SCC25 and SAS cells, LowCa induced significant change of cell shape (Fig. 8a, c). Although with an increase in the averaged aspect ratio of OECM1 cells, the difference was not significant (Fig. 8b). Mesenchymal cellular phenotypes are associated with cellular polarization, degradation of extracellular matrix (ECM), and directed migration [4]. The results demonstrated that alteration of cellular phenotypes could be induced when cancer cells were cultured with calcium deprivation.

### 1.8 Invadopodia induction by calcium deprivation in HNSCC is reversible

**Fig. 9:**
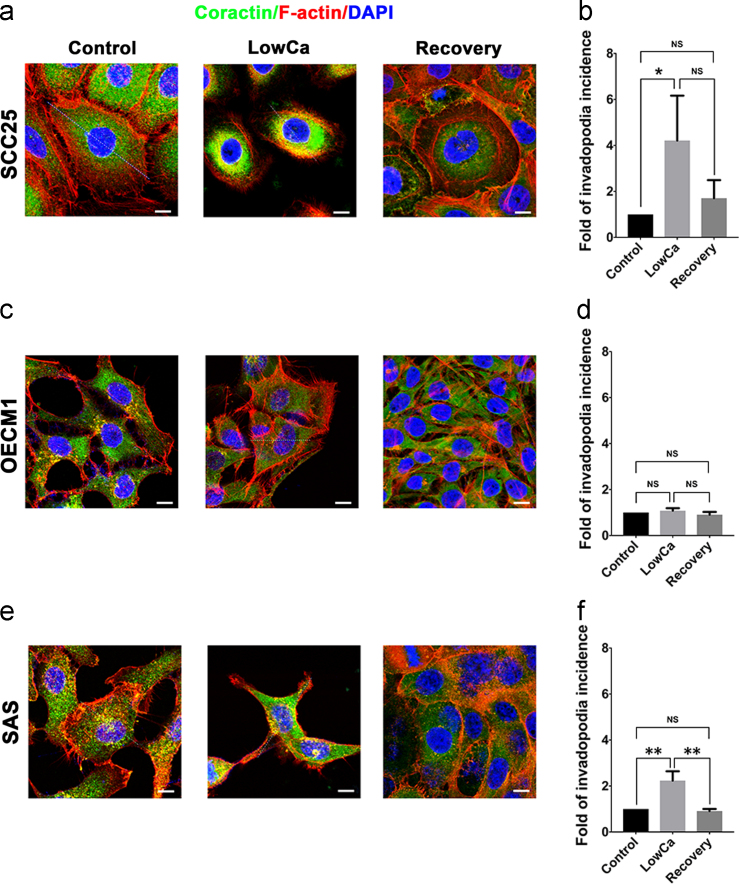
Invadopodia induction by calcium deprivation in HNSCC is reversible. Representative immunofluorescent imaging of coexpression of cortactin (green) and F-actin (red) of the control, LowCa, and recovery groups in (a) SCC25, (c) OECM1, and (e) SAS cells. The quantitative results demonstrated the incidence of invadopodia formation in the control, the LowCa, and the recovery groups of (b) SCC25, (d) OECM1, and (f) SAS cells. (scale bar: 10 μm; NS: no significance; **p<0.01). (For interpretation of the references to color in this figure legend, the reader is referred to the web version of this article.)

Invadopodia were characterized by coexpression of cortactin and F-actin [5], [6]. Using these markers, increased invadopodia formation was identified in cell periphery when calcium was deprived in both SCC25 cells and SAS cells, and these effects were reversible after calcium repletion (Fig. 9a, b, e, f). When calcium concentration increased in the LowCa group, formation of invadopodia decreased. For OECM1 cells that already had high invadopodia incidence, invadopodia formation was not enhanced by LowCa (Fig. 9c, d). These findings were in accordance with the results of aspect ratios (Fig. 8) [1]. In addition, induced invadopodia formation was reversible when calcium concentrations were adjusted. These data altogether provided evidences to support that both morphological alteration and invadopodia formation of HNSCC cells could be induced in our system.

### 1.9 LowCa reduced the membranous expression of E-cadherin in HNSCC cells

**Fig. 10:**
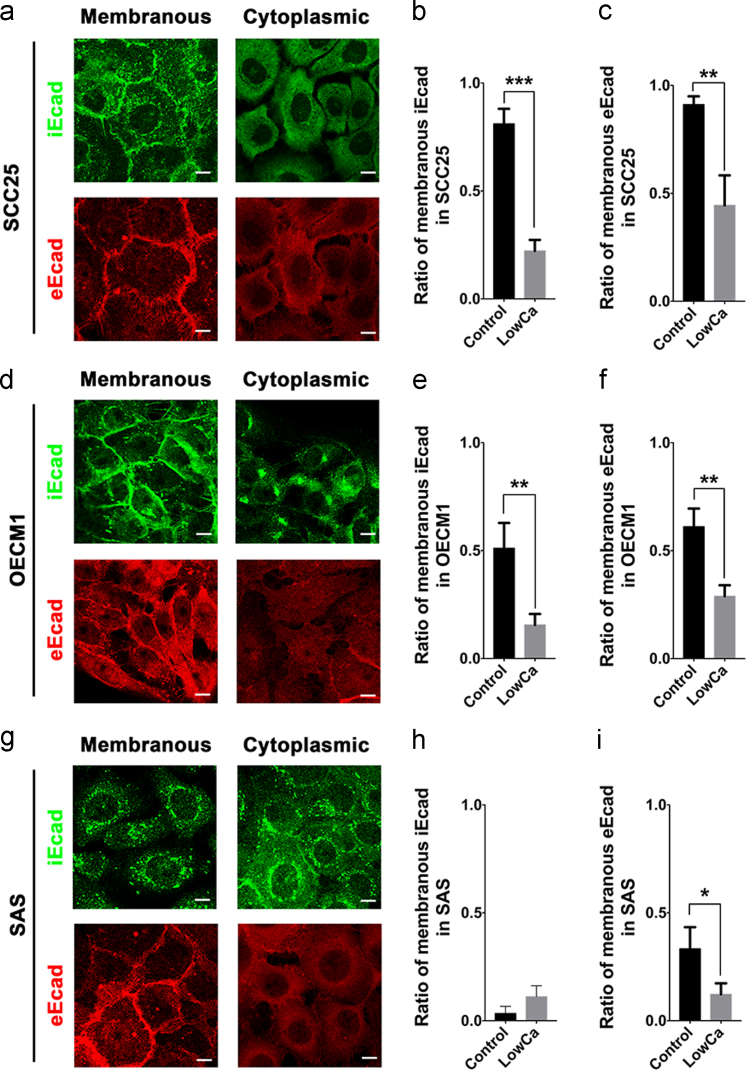
Proportion of membranous expression of E-cadherin in HNSCC cells with calcium deprivation. Representative membranous and cytoplasmic expression of iEcad and eEcad in (a) SCC25, (d) OECM1, and (g) SAS cells. Quantitative results of the change of membranous iEcad in the control and LowCa groups of (b) SCC25, (e) OECM1, and (h) SAS cells. Quantitative results of the change of membranous eEcad in the control and LowCa groups of (c) SCC25, (f) OECM1, and (i) SAS cells. (scale bars in a, d, g: 10 μm; *p<0.05; **p<0.01; ***p<0.001).

Loss of membranous E-cadherin expression is associated with high grades and advanced stages of cancer [7]. To verify the change of E-cadherin expression patterns, E-cadherin was classified as membranous and cytoplasmic based on expression of selective immunofluorescent staining of the intracellular domain (iEcad) and the extracellular domain of E-cadherin (eEcad), respectively. In addition to identifying iEcad and eEcad translocation from membranes into cytoplasm in a single cell [1], the cells expressing membranous iEcad and eEcad also decreased after calcium deprivation in SCC25 and OECM1 cells (Fig. 10a–f). In SAS cells, only reduction of cells with membranous eEcad was detected (Fig. 10g, h, i). These data showed that most HNSCC cells lost membranous E-cadherin expression treated by LowCa.

### 1.10 Recapitulation of tumor satellite formation in the 3D culture system with compatible cellular features of cancer specimens

**Fig. 11:**
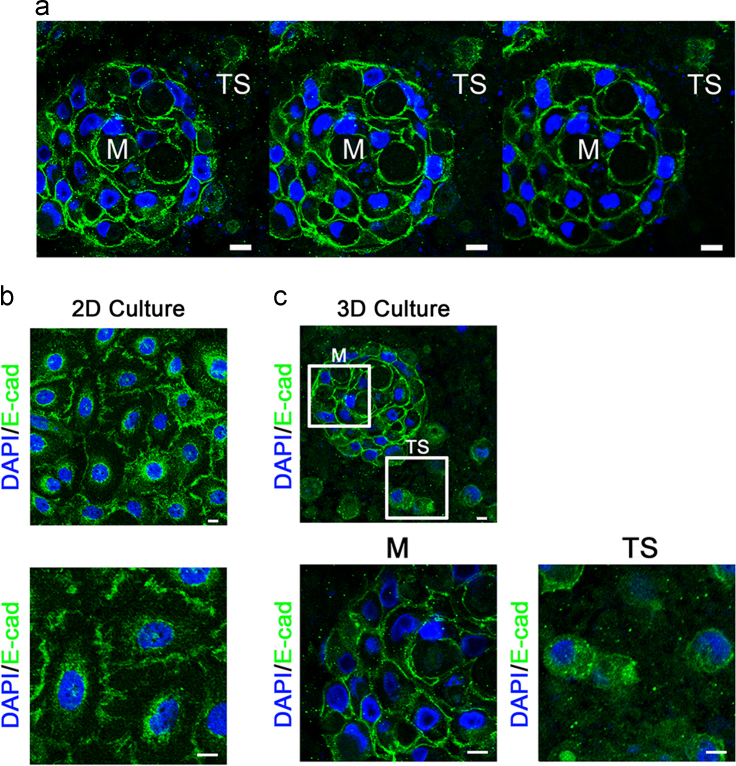
Immunofluorescent staining of E-cadherin in 2D and 3D *in vitro* culture systems. (a) The serial sections of cancer cell masses and satellites *in situ* from the 3D culture. The serial sections demonstrated that E-cadherin expression shifted from membranous staining in the main tumor mass (M) to cytoplasmic staining in the tumor satellites (TS). (b) The expression pattern of E-cadherin in the original HNSCC cells cultured in a conventional 2D manner. The lower panel is the magnification of the upper panel. (c) Serial sections taken through the 3D cultures were shown for E-cadherin immunolabeling of the cell masses and satellites *in situ.* The lower panels are the magnification of indicated areas in the upper panel (the scale bar: 10 µm).

The serial sections of 3D collagen system were performed to identify the tumor mass and satellite cells *in situ*. By immunofluorescent staining, membranous E-cadherin predominantly expressed in the tumor mass whereas cytoplasmic E-cadherin was demonstrated in adjacent dissociated tumor satellites (Fig. 11a). In the 2D culture system, most cells had membranous E-cadherin expression (Fig. 11b), which was compatible with the expression patterns demonstrated by the tumor mass in 3D culture systems (Fig. 11b, c). On the contrary, when tumor satellites were induced in the 3D culture system, E-cadherin expression changed from membranous patterns to cytoplasmic patterns (Fig. 11c). It was compatible with *in vivo* pathological findings that cytoplasmic E-cadherin expression was found in the tumor satellites [1]. The results demonstrated the capacity of this 3D culture system of recapitulating the phenomena of tumor satellite formation of cancer cells.

### 1.11 The proposed model of tumor satellite formation in cultured cancer cells

**Fig. 12:**
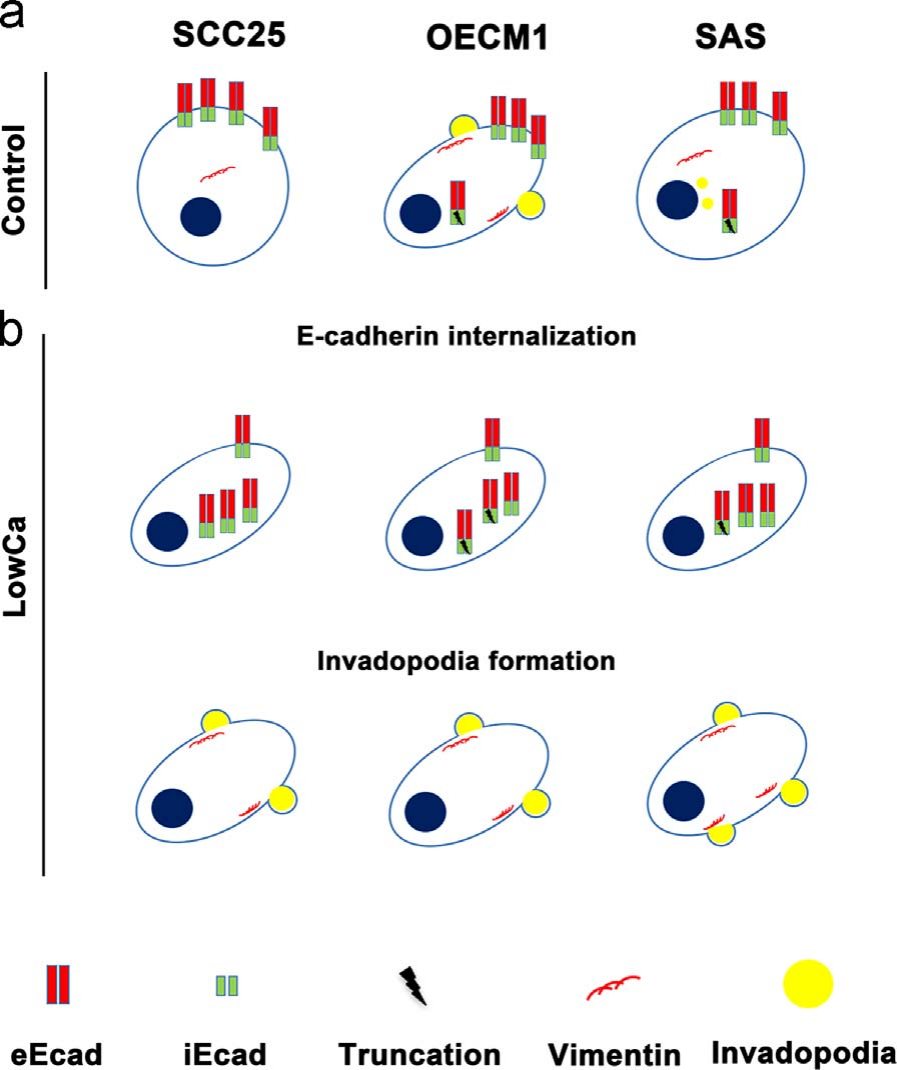
The proposed model of tumor satellite formation induced by calcium deprivation. (a) Parental HNSCC tumor cells have distinct morphology and the characteristics of iEcad, eEcad, vimentin, and invadopodia. (b) When induced by calcium deprivation (LowCa) to form tumor satellites, the cellular morphology changes. The specific cytological characteristics pertinent to tumor satellite formation in different HNSCC tumor cells are depicted.

According to these results, a model is proposed for tumor satellite formation of HNSCC cultured in the 3D system with calcium deprivation. The intrinsic characteristics of tested cell lines including original morphology, E-cadherin expression, and invadopodia formation are different (Fig. 12a). E-cadherin internalization is beneficial for cellular dissociation while vimentin translocation is important for invadopodia formation, both can be induced by the current system [1]. For the cancer cells such as SCC25 with incomplete EMT process that are characterized by loss of E-cadherin and gain of vimentin, the impact of LowCa is more effective than others. The effect is beneficial for the formation of tumor satellites (Fig. 12b). Even for other HNSCC cells with complete EMT status, LowCa still enhances E-cadherin internalization and invadopodia formation than controls, which facilitates tumor satellite formation and increases their ability (Fig. 12b).

## 2 Experimental design, materials and methods

### 2.1 Establishment of the *in vitro* three-dimensional culture system

The human cancer cell lines with different malignant potential including SAS cells [8], OECM1 cells [9], SCC25 cells [10], CT26 cells [11], and A375 cells [12] were enrolled in this study. Type I collagen from rat tails (BD Biosciences) was used to fabricate the three-dimensional (3D) collagen scaffolds. It was mixed with reconstitution buffer to form the collagen solution first (3.5 mg/mL in 0.02 N acetic acid). The media specific for each cancer cell line was then mixed with the collagen solution (Medium: Collagen Solution = 1:9). The media used for SCC25 cells was DMEM/F12. The RPMI was for OECM1 cells and CT26 cells, while DMEM was for SAS cells and A375 cells. The collagen gel was first coated on the plate as the bottom gel [13]. After gelation of the bottom gel, micromass pellet culture was prepared from each cell line and placed on the center of the bottom gel. 10% 0.26 M NaHCO_3_ was added in the collagen-medium mixture for preparing the top gel after polymerization [13]. Then a sandwich-like three-dimensional culture system with cancer cells embedded was established to mimic *in vivo* environment. In the controls, the calcium concentration was 1.05 mM. For the groups with calcium deprivation (LowCa), the calcium concentration was 0.15 mM. The concentration was determined by previous reports and testing of serial dilution [2], [3], [14]. For the recovery assays, the media of LowCa groups were replaced with the media with normal calcium concentrations 2 days later.

### 2.2 Imaging and analyses of tumor invasion patterns

The cellular behaviors were recorded by time-lapse microscopy and confocal microscopy (Zeiss Axiovert 200M Inverted Confocal Microscope). The strand pattern of tumor invasion was defined as the advancing edge with cells in strands, while the satellite pattern of tumor invasion was defined as dissociated tumor cells with intervening normal tissue at the tumor and non-tumor interface [15], [16], [17], [18], [19]. The variables of invasion patterns were compared such as the incidence, cell migratory trajectory, accumulated distances, and tumor-satellite distance. The accumulated distance was defined as the whole distance of satellite cell movement during trajectory tracking, and the tumor-satellite distance (TSD) was defined as the longest straight distance between the parental tumor and the satellite cells [18], [20].

### 2.3 Immunofluorescence and imaging

The cells were harvested from each group after culture. The procedure of immunofluorescent staining followed the standard protocol previously described [21], [22], [23]. The primary antibodies used in the study included E-cadherin (extracellular domain; Santa-cruz7870, 1:50), E-cadherin (intracellular domain; BD 610182, 1:50), Vimentin (Abcam), F-actin (Invitrogen), and Cortactin (Milipore). Confocal images were obtained by Carl Zeiss LSM780/570 confocal microscopes. The ratio of cells with membranous E-cadherin in the sampling field was defined as the number of cells expressing membranous E-cadherin divided by the total number of cells.

### 2.4 Assays of cell migration, proliferation, and viability

For cell migration assays, the cells were dispersed equally and cultured with either control or LowCa medium, respectively. Cell migration was tracked by time-lapse microscopy, and quantitatively analyzed by the Metamorph software including cell migration distance and displacement. For proliferative assays, immunostaining of cells was performed as the method mentioned above using the antibodies of PCNA (Abcam) and Ki-67 (BD Bioscience). The secondary antibodies were applied for visualization and recording. The proliferative rate was defined as the ratio between the number of cells showing positive proliferative markers and the total cell numbers. To further elucidate the change of proliferation and viability of cultured cells, the MTT assay was performed [24]. Suspended cells were immersed with 10 μl 3-(4,5-dimethylthiazol-2-yl)-2,5-diphenyltetrazolium bromide solution (MTT, Sigma-Alderich; 5 mg/ml; 37 °C, 3 h). The MTT solution was then replaced with 100 μl dimethyl sulfoxide (Sigma-Alderich) for formazan reaction. The absorbance was recorded by a spectrophotometer at 570 nm [24]. The relative MTT value of each experimental group was normalized with controls.

### 2.5 Western blotting

The cell lysates were harvested after culture, separated by SDS-PAGE, and then transferred. The antibodies used included HIF1α (Abcam), prolyl hydroxylase 2 (PHD2) (Abcam), and beta-actin (Abcam); The results were detected using horseradish peroxidase-conjugated secondary antibodies (Jackson Biotechnology) and developed by the ECL reagent (Millipore). Digital images of immunoblots were obtained with a UVP and analyzed using the image analysis program, Image J [25].

### 2.6 Immunofluorescent staining of E-cadherin in the serial sections of 3D culture system

The 3D collagen cultures were fixed and embedded in 1% agarose gels. The whole agarose block was incubated with sucrose solutions with gradient concentrations. Five-micrometer frozen sections of tumor tissue in O.C.T. were prepared. The sections were then incubated with the primary antibody (E-cadherin: BD610158) overnight at 4 °C, and subsequently with the secondary antibody (Alexa Fluor®) for 1 h at RT. The nucleus was counterstained with DAPI (Sigma). The images were acquired by confocal microscopy for analyses.

### 2.7 Statistical analysis

All measurements were compiled from three or more independent experiments for each condition. The data were compared using two-sided Student's *t*-test and chi-square test. *p*<0.05 or less is considered statistically significant.
