# Supplementary material for: The data of establishing a three-dimensional culture system for in vitro recapitulation and mechanism exploration of tumor satellite formation during cancer cell transition
Source: Data Brief. 2017 Sep 28;15:545–61. doi: 10.1016/j.dib.2017.09.053 (PMC5651497; doi:10.1016/j.dib.2017.09.053)
Supplement: Supplementary file 1 — Supplementary material [file mmc1.pdf]

Conflict of interest

The authors declare no potential conflicts of interest.
